# Metabolic syndrome in the era of COVID-19 outbreak: impact of lockdown on cardiometabolic health

**DOI:** 10.1007/s40618-021-01563-y

**Published:** 2021-05-26

**Authors:** R. S. Auriemma, R. Pirchio, A. Liccardi, R. Scairati, G. Del Vecchio, R. Pivonello, A. Colao

**Affiliations:** 1 Dipartimento di Medicina Clinica e Chirurgia, Sezione di Endocrinologia, Università “Federico II” di Napoli, Naples, Italy; 2 UNESCO Chair for Health Education and Sustainable Development, Federico II University, Naples, Italy

**Keywords:** COVID-19 pandemics, Metabolic syndrome, Cardiometabolic risk, Restrictions, Changes in lifestyle, Social isolation

## Abstract

**Purpose:**

COVID-19 pandemics and cardiometabolic health are mutually interconnected. Chronic metabolic diseases are known risk factors for increased mortality after SARS-CoV-2 infection. In turn, COVID pandemics imposed sudden changes in lifestyle and social isolation with consequent potential cardiometabolic sequelae. The present study aimed at investigating the impact of changes in lifestyle and social life on metabolic profile in hyperprolactinemic or osteoporotic patients without pre-existing cardiometabolic diseases at the time of COVID-19.

**Methods:**

The primary study outcome measurement was the prevalence of obesity, arterial hypertension, impaired glucose tolerance (IGT) or diabetes mellitus (DM), dyslipidemia and metabolic syndrome after COVID-19 outbreak. Seventy-four patients (21 men and 53 women, aged 51.8 ± 17.8 years) were admitted to the outpatient clinic of the Neuroendocrine Disease Unit at University “Federico II” of Naples, Italy, as per their routine clinical practice because of tumoral and non-tumoral hyperprolactinemia in 52 patients (70.3%), and osteoporosis/osteopenia in 22 (29.7%). Among female patients, 25 (47.2%) were at menopausal age.

**Results:**

At the end of lockdown, prevalence of obesity (from 37.8% to 51.3%, *p* < 0.0001), dyslipidemia (from 28.4 to 48.6%, *p* = 0.003) and metabolic syndrome (from 14.9 to 27%, *p* < 0.0001) significantly increased compared to pre-COVID evaluation. No significant change was found in the prevalence of arterial hypertension and IGT/DM.

**Conclusion:**

SARS-CoV-2 outbreak has led to a rapid increase in the prevalence of metabolic syndrome, potentially contributing to the increased COVID-19 related mortality.

## Rapid communication

Between March, the 9th 2020 and May, the 4th 2020 special restrictions with a complete lockdown have been imposed by the Italian Government to limit the outbreak of COVID-19 pandemics in Italy. As a result, Italian people have been required to stay home for approximately 2 months; any physical exercise in public sites, such as gyms and parks, has been forbidden and most professional activities have been turned into a smart working modality, with the exception of employers for essential services, including hospitals and other healthcare, utilities such as electricity and water supply, law enforcement and firefighting, and mostly food services. Such restrictions have been importantly effective in reducing viral spread, with infection and mortality rates progressively decreasing up to reach a safer condition, to stop the lockdown and to restart most routine working activities and social life. On the other side, these restrictive measures negatively influenced the everyday life, not only for elderlies considered to be at high risk of COVID-19 infection mainly if affected with chronic cardiometabolic diseases, but also for young healthy people.

However, during the approximately 60 days of lockdown Italians have changed not only social and public behavior, but mainly their lifestyle. At one side, in the vast majority of cases, routine physical exercise has been strongly limited to a few-minute domestic in-house fitness or totally abandoned, and most people have adopted a sedentary behavior. On the other side, time availability and rediscovery of familial conviviality exerted a negative impact also on dietary behavior, as demonstrated by the wide use of home-made food mainly prepared by fat sources (fresh meat, eggs, butter, long-life milk), or by whole white flour and sugar, such as pizza, cakes, pasta and bread [1]. In turn, the use of fresh healthy food, mainly including cereals, fish, fruits and vegetables, has been reduced despite its permanent availability at food stores [1], thus leading to a limited adherence to the Mediterranean diet, known to exert strong cardioprotective effects [2]. Note to worth, consequences of such changes in lifestyle and nutritional habits are even more alarming in patients with pre-existing obesity, diabetes mellitus and metabolic syndrome (MetS), which in turn have been demonstrated to be predisposed to higher risk of mortality following SARS-CoV-2 infection [3]. Social isolation and loneliness, known to be associated with increased mortality risk mainly among elderlies, has resulted in notable changes in patterns of daily living, mainly timing of meals and sleep, and altered circadian biology, which in turn might severely impact cardiometabolic health [4]. This evidence has raised the questions of whether these changes might impair metabolic profile also in healthy people not affected by cardiometabolic diseases at the time of COVID-19 outbreak, and whether metabolic impairment might be an aftermath, and not only a risk factor, of COVID-19 disease.

**Fig. 1:**
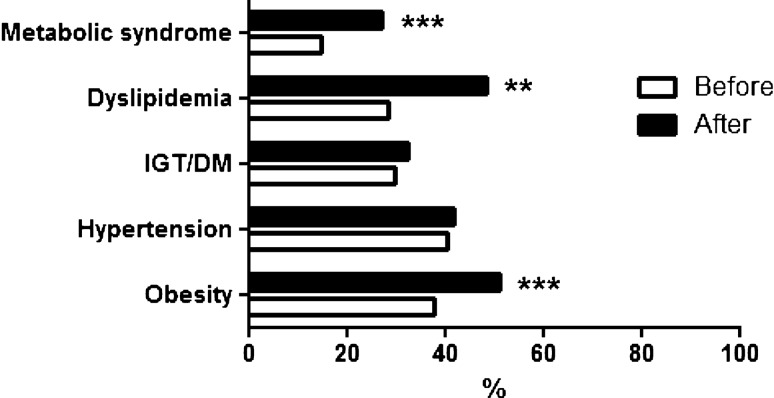
Changes in the prevalence of metabolic syndrome, dyslipidemia, impaired fasting glucose/diabetes mellitus, arterial hypertension and obesity after COVID-19 outbreak. Consequently to the restrictive measures required for COVID-19 pandemics and total lockdown, prevalence of metabolic syndrome, dyslipidemia and obesity significantly increased in the present cohort of hyperprolactinemic or osteoporotic patients without pre-existing metabolic disorders before COVID-19 outbreak

By the end of lockdown up to July, the 30th 2020, 74 patients, including 21 men (28%) and 53 women (72%), aged 51.8 ± 17.8 years, were consecutively admitted to the outpatient clinic of the Neuroendocrine Disease Unit at University “Federico II” of Naples as per their routine clinical practice because of tumoral and non-tumoral hyperprolactinemia in 52 patients (70.3%), and osteoporosis/osteopenia in 22 (29.7%). Among female patients, 25 (47.2%) women were at menopausal age. Based on power calculation and sample size analysis, a total of 66 patients were required for a statistical power of 80% at 5% significance set. Based on age quartiles, patients were stratified as group 1 (16–38.25 years, 19 patients), group 2 (38.36–50 years, 19 patients), group 3 (50.1–67.75 years, 19 patients), group 4 (67.76–90 years, 17 patients). As shown in Fig. 1, at last follow-up before COVID-19 pandemics (within 3–6 months), 28 (37.8%) were obese, 30 (40.5%) had arterial hypertension, 22 (29.7%) impaired glucose tolerance (IGT) or overt diabetes mellitus (DM), and 21 (28.4%) dyslipidemia. According to the International Diabetes Federation consensus criteria [5], overall MetS was found in 11 patients (14.9%, Fig. 1).

Throughout the lockdown period, all patients maintained treatment schedules previously established on the basis of their pre-existing endocrine disease and none suddenly withdrew medical therapies for concomitant cardiometabolic diseases. None of these patients developed SARS-CoV-2 infection.

At the evaluation after the lockdown period (Fig. 1), 38 patients (51.3%) were obese (*p* < 0.0001), 31 (41.9%) had arterial hypertension (*p* = 0.995), 24 (32.4%) IGT or DM (*p* = 0.859), and 36 (48.6%) had dyslipidemia (*p* = 0.003). New anti-hypertensive drugs, glucose lowering drugs and lipid lowering drugs were added in 1 (3%), 5 (20.8%) and 12 (33.3%) patients, respectively. Prevalence of arterial hypertension was significantly higher in group 3 (*p* < 0.0001) and group 4 (*p* < 0.0001) as compared to group 1, and in group 2 (*p* = 0.002) as compared to group 3. Prevalence of IGT/DM was significantly higher (*p* = 0.024) in group 2 as compared to group 3. No significant difference was found in the prevalence of dyslipidemia among age groups. Overall MetS was found in 20 (27%, *p* < 0.0001) (Fig. 1). Prevalence of MetS was significantly higher in group 3 (*p* < 0.0001) and group 4 (*p* = 0.043) as compared to group 1, and in group 2 (*p* = 0.007) compared to group 3.

In our experience, the significant and rapid worsening of cardiometabolic health seen in our patients reflects changes in lifestyle, nutritional habits and social isolation induced by the forced lockdown due to COVID-19 pandemics and affects not only patients with pre-existing metabolic alterations but also those with documented metabolic health before COVID-19. In other words, SARS-CoV-2 outbreak has led to a rapid MetS outbreak, which in turn might furtherly alter immunometabolism and promote chronic systemic inflammation, thus exacerbating hyperinflammation associated with SARS-CoV-2 infection and contributing to COVID-19 increased mortality [6]. Prolonged and sustained stress at one side, and social isolation on the other side have produced detrimental effects on cardiometabolic health. These findings deserve utmost consideration, taking into account that the impact of isolation and loneliness on health and mortality are reportedly of the same order of magnitude as other risk factors in COVID-19 patients such as arterial hypertension, obesity, and smoking [7]. Future research will elucidate the role and the burden of COVID-19 pandemics on long-term cardiometabolic sequelae.
